# Feasibility of avian antibodies as prophylaxis against enterotoxigenic escherichia coli colonization

**DOI:** 10.3389/fimmu.2022.1011200

**Published:** 2022-10-19

**Authors:** Kyle Brumfield, Hyesuk Seo, Nnebuefe Idegwu, Chad Artman, Laura Gonyar, James Nataro, Weiping Zhang, David Sack, James Geyer, Julius Goepp

**Affiliations:** ^1^ Maryland Pathogen Research Institute, University of Maryland, College Park, MD, United States; ^2^ University of Maryland Institute for Advanced Computer Studies, University of Maryland, College Park, MD, United States; ^3^ Department of Therapeutics Development, Scaled Microbiomics, Hagerstown, MD, United States; ^4^ Department of Pediatrics, University of Virginia, Charlottesville, VA, United States; ^5^ Department of Pathobiology, University of Illinois Urbana-Champaign, Urbana, IL, United States; ^6^ Bloomberg School of Public Health, Johns Hopkins University, Baltimore, MD, United States; ^7^ Institute for Rural Health Research, University of Alabama, Tuscaloosa, AL, United States

**Keywords:** avian immunoglobulins, IgY, enterotoxigenic E. coli (ETEC), travelers diarrhea, prophylaxis, polyclonal antibodies (PAbs)

## Abstract

**Background:**

This research aims to evaluate the feasibility of using avian immunoglobulins (IgY) raised against adhesion factors of enterotoxigenic *Escherichia coli* (ETEC) as prophylaxis of diarrheal illness caused by these pathogens. ETEC requires adhesion to human intestinal epithelial cells as a primary step in establishing enteric infection. Therefore, inhibition of adhesion may prevent such infections and reduce clinical burdens of diarrheal illness.

**Methods:**

IgY samples were prepared from eggs of hens immunized with an adhesin-tip multiepitope fusion antigen (MEFA), developed against nine adhesin tip epitopes derived from clinically relevant ETEC strains. The resulting IgY was evaluated for its ability to inhibit adhesion of ETEC to cell-surface targets. Potential impacts of anti-MEFA IgY on growth of both pathogenic and commensal *E. coli* isolates were also evaluated.

**Results:**

Enzyme linked immunosorbent assay (ELISA) titers were achieved for IgY targeting each of the nine individual epitopes included in the adhesin-tip MEFA. Furthermore, anti-MEFA titers exceeding 1:2^19^ were sustained for at least 23 weeks. All ETEC strains used in design of the adhesin-tip MEFA, and five of five clinical ETEC strains were significantly (P < 0.05) inhibited from adhesion to mammalian cells in culture.

**Conclusions:**

These findings demonstrate that IgY targeting ETEC adhesin-tip MEFA have the potential to disrupt *in vitro* adherence of ETEC. A formulation containing adhesin-tip MEFA IgY can be considered a potential candidate for *in vivo* evaluation as prophylaxis of diarrheal diseases. Animal studies of this formulation are planned.

## Introduction

Enteric diseases causing severe diarrhea threaten survival of children and are a source of considerable morbidity in low- and middle-income countries (LMIC) (1). Enterotoxigenic *Escherichia coli* (ETEC), heterogeneous strains of *E. coli* that produce enterotoxins, are predominant bacterial pathogens associated with acute gastroenteritis (AGE) in humans (1). Various strains of ETEC are also common etiopathogens in travelers’ diarrhea (TD), affecting both children and adults traveling from industrialized nations to LMIC and military and diplomatic personnel stationed in endemic regions (2, 3).

To express primary virulence factors and establish infection associated with AGE, ETEC bacteria must first adhere to mucosal epithelial cells in the lumen of the small intestine (4). Crucial ETEC virulence factors are cell-surface adhesins, including colonization factor antigens (CFAs) and coli surface antigens (CSs), as well as heat-labile (LT) and heat-stable (ST) enterotoxins delivered into intestinal epithelial cells after mucosal colonization, which promote cyclic nucleotide production and ultimately trigger fluid efflux from the circulation into the ileal lumen, resulting in watery diarrhea (4).

The quest for broadly effective anti-ETEC vaccines long focused on identification and antigenicity of the myriad adhesin molecules expressed by various clinically important ETEC strains; however, this approach is vulnerable to antigenic drift and emergence of molecularly distinct CFs and CSs (5). A broader mechanistic approach has seen the emergence of multivalent protein-based ETEC vaccine candidates that induce antibodies neutralizing both LT and STa toxins as well as antibodies inhibiting adherence of multiple ETEC adhesins, which have been shown to be protective against ETEC toxin-mediated diarrhea in a pig challenge model (6). Further, such multivalent vaccines reduced colonization by the most virulent ETEC strain, H10407, in a rabbit intestinal colonization model (6). Despite these recent advances, however, no licensed vaccine against ETEC is available, though several are in early-stage clinical trials (7, 8).

An appealing alternative to vaccine-derived immunization is to provide passive immunity prior to exposure by delivering protective polyclonal antibodies directly to the small intestinal mucosa, the sites of ETEC action. This would be especially useful when persons are at high risk for a relatively brief time, such as travelers, though there may be other groups where such prophylaxis will be desirable. Previous work has shown that bovine anti-ETEC immunoglobulins (IgG) delivered in bovine colostrum can reduce incidence and volume of diarrheal stools in human volunteers challenged with a single strain of ETEC (H10407), and at least one commercial product employing IgG against ETEC is available (9, 10).

Immunoglobulin Y (IgY), the primary circulating antibody of avians, extracted from the yolks of specially immunized hens, offers certain advantages over mammalian antibodies. IgY fails to fix mammalian complement and does not bind to mammalian F_c_ receptors, potentially reducing risk of undesired complement activation (11). Target-specific IgY can be developed quickly at low cost by immunization of a small number of hens, permitting rapid iteration of target antigens to select a final optimized form. Hens may be immunized with multiple antigens simultaneously, resulting in polyclonal IgY targeting multiple steps in a pathogenic process (12). Studies in agricultural and laboratory settings suggest that IgY targeting animal enteropathogens is effective at both prevention and mitigation of diarrheal symptoms (13).

Given the absence of widely available and effective vaccines for ETEC, high mortality associated with diarrhea in LMIC, and evidence that passive immunization with IgY is effective in animal models, we set out to evaluate the feasibility of producing IgY targeting multiple common ETEC adhesins as a potential diarrheal prophylactic in humans. Furthermore, we examined potential impacts of the resulting IgY on growth of various pathogenic and commensal *E. coli* isolates.

## Materials and methods

### IgY production, extraction, and characterization

#### Bacterial strains and IgY targets

An adhesin-tip multi-epitope fusion antigen (MEFA), which integrates epitopes from adhesin tips or adhesive subunits of nine common ETEC adhesins (CFA/I, CS1, CS2, CS3, CS4, CS5, CS6, CS21, and EtpA) to create an adhesin-tip MEFA peptide was previously described in the literature (4). Briefly, the adhesin-tip MEFA gene was designed *in silico* using the CFA/I tip CfaE gene (*cfaE*) as the backbone, with nucleotide fragments encoding the most antigenic epitopes of the remaining eight adhesin tip or adhesive subunits; the resulting product was synthesized and subsequently cloned into a vector and expressed in an *E. coli* BL21 expression system (GE Healthcare, Piscataway, NJ); bacterial proteins were then extracted using bacterial protein extraction reagent (B-PER; Pierce, Rockford, IL), and subsequently refolded using a protein refolding kit (Novagen, Madison, WI) by following manufacturer’s protocol(4). The refolded MEFA protein was suspended in solubilization buffer (50 mM CAPs) supplemented with 0.3% N-lauroylsarcosine and 1mM ethylenediamine tetraacetate (EDTA). Refolded adhesin-tip MEFA proteins were collected with centrifugation at 12,000 rpm for 10 min at 4°C, measured for protein concentration, aliquoted, and stored at −80°C until use for anti-ETEC adhesin IgY preparation as detailed below(4). Adhesins and ETEC isolates from which they were derived are shown in **Table 1** (4).

**Table 1:** MEFA development strains.

Strains	Relevant properties(4)
H10407	O78:H11; CFA/I, LT, STa
THK38/pEU405	CS1
DH5α/pEU588	CS2
E116 (E19446)	CS3, LT, STa
E106 (E11881/9)	CS4/CS6, LT, STa
UM 75688	CS5/CS6, LT, STa
2423 ETP98066	CS6, LT, STa
JF2101	CS21/EtpA/EatA, STa
JF2318 ETP050008	EtpA, STa

ETEC strains used in development of ETEC adhesin-tip MEFA, as described previously (4). CFA: Colonization factor antigens; CS: Coli surface antigens; LT: ETEC heat-labile toxin; ST: ETEC heat-stable toxin; etpA: outer membrane protein adhesin.

#### Immunization of laying hens

Eight Commercial White Rock and Rhode Island cross-bred, sexlink hens (Pinola Hatchery, Shippensburg, PA, USA) were housed in a purpose-built henhouse permitting segregation of paired hens. Hens were acclimated for two weeks prior to immunization at ambient temperatures on a 12-hour light/dark cycle on *ad libitum* water and a commercial diet (Martin’s Layer Mash 16%, Martin’s Elevator, Inc., Hagerstown, MD, USA). Protocols for hen maintenance and immunization were approved by the Scaled Microbiomics, LLC Animal Use and Care Committee (approval number 19-01-TD).

Adhesin-tip MEFA protein was added to avian adjuvant Montanide ISA 70 VG (Seppic, Inc., La Garenne-Colombes, France) and phosphate-buffered saline (PBS) mixture (7:3 v/v) in a high-shear blender to a final concentration of 100 µg/mL of MEFA protein, and filter sterilized using a 0.2 µm pore size polyethersulfone filter membrane (VWR International, Radnor, PA, USA). Sterility was confirmed by absence of visual growth after inoculating 25 µl of antigenic mixtures into fastidious BBL™ Schaedler broth with Vitamin K_1_ (Becton Dickinson, Sparks, MD, USA) and incubating for 48 without aeration at 37 °C. On day 1 of hen immunizations, four hens were injected with 0.5 ml in each breast muscle, delivering a total of 100 µg of ETEC adhesin-tip MEFA per hen. Booster injections were administered in an identical fashion on days 14 and 28 of hen immunizations. Two additional hens designated “sham injected”, received 0.5 ml of PBS and adjuvant only, prepared as previously described without MEFA protein, in each breast during immunizations (days 1, 14, and 28). A final hen pair was used for control and received no immunizations, designated “unimmunized.”

#### IgY extraction and concentration

Two eggs were collected weekly from each hen pair beginning one day prior to the first immunization injections; after demonstration of detectable titers at Week 4, all eggs were collected from each hen pair. IgY was extracted from yolks using polyethylene glycol (PEG), as described elsewhere (14), with the following modifications. Briefly, yolks were pooled, and lipid content was removed by centrifugation (13,000 x g for 20 min at 4°C) using PEG 6000 at consecutively increasing concentrations (3.5, 8.5, and 12% w/v; Alfa Aesar, Haverhill, MA, USA). The resulting precipitate was resuspended in PBS and dialyzed against sodium chloride (0.1% w/v) for 16 hours and PBS for an additional three hours using Spectra/Por 4 standard RC dialysis tubing (12-14 kD; Spectrum Laboratories, Inc, Rancho Dominguez, CA). The dialyzed water-soluble fraction (WSF) containing IgY was stored at -20°C until further analysis (< 2 weeks). Material from pooled yolks harvested between weeks 7 and 11 was fully characterized as follows and then used in all subsequent experiments.

Total protein concentrations of WSF were determined by bicinchoninic acid (BCA) method kit (Thermo Fisher Scientific, Rockford, IL, USA), following manufacturer’s specifications. Absorbance values were read at 490 nm using THERMOmax microplate reader (Molecular Devices, Sunnyvale, California, USA), and standard curve showed linear behavior (R^2^ = 0.99) over seven serial 1:2 dilutions (0.06-2 mg/ml) of the bovine serum albumin protein standard set (Thermo Fisher Scientific, Waltham, MA, USA).

### Sodium dodecyl sulfate-polyacrylamide gel electrophoresis

To determine purity of yolk-derived IgY, SDS-PAGE was conducted under reducing conditions using 12% polyacrylamide gel (NuSep Inc., Germantown, MD, USA) with a Novex Mini-Cell (Invitrogen, Carlsbad, CA, USA). Briefly, purified WSF samples were diluted 1:10 in PBS, mixed with equal volume of sample buffer, and denatured for 5 min at 100°C. A total of 20 μl of sample/buffer mixture was loaded into each well, and protein bands were visualized with Protein Fixative (Ward’s Science, Rochester, NY, USA), as recommended by the manufacturer. Gels were imaged using a standard camera.

### Enzyme-linked immunosorbent assay: IgY in MEFA immunized hens’ eggs

To determine the IgY response to immunization with the adhesin-tip MEFA itself, IgY titers against the adhesin-tip MEFA were measured by indirect noncompetitive ELISA, as reported previously (15–17), with slight modifications. Briefly, 96-well flat bottom microtiter plates were coated with 400 ng of MEFA diluted in 5x ELISA Coating Buffer (BioLegend, San Diego, CA) to a final MEFA concentration of 4 ng/µl and incubated overnight at 4°C. The coating solution was aspirated, and plates were washed three times with commercial ELISA wash buffer (Thermo Fisher Scientific, Waltham, MA, USA) and blocked at 4°C overnight (16 h) using 5% (w/v) nonfat milk (VWR International, Radnor, PA, USA) dissolved in PBS (VWR International, Radnor, PA, USA). Following aspiration of the blocking solution, plates were incubated with three serial 1:2 dilutions (2^20^ to 2^22^) of sample anti-MEFA IgY (or control Unimmunized IgY) in blocking buffer (5% w/v nonfat milk) for one hour at room temperature (23-25°C). Primary antibody solutions were aspirated, and plates were washed 3 times with commercial ELISA wash buffer. Bound anti-MEFA IgY was detected by horseradish peroxidase (HRP)-conjugated goat anti-chicken secondary antibody (ImmunoReagents, Inc., Raleigh, NC, USA diluted (1:2,500) in blocking buffer (5% w/v nonfat milk) and incubated at room temperature for one hour. Plates were washed five times using commercial ELISA wash buffer (Thermo Fisher Scientific, Waltham, MA, USA) and visualized using 3,3’-5,5’-tetramethylbenzidine (TMB; VWR International, Radnor, PA, USA), following addition of an equivalent volume of 2N sulfuric acid. Optical density (OD) was measured on a THERMOmax microplate reader (Molecular Devices, Sunnyvale, California, USA) at 450 nm (OD_450_). Antigen-specific IgY titer was defined as the maximum dilution multiple of the sample with an OD_450_ value that was 2.1 times greater than the unimmunized control.

### Enzyme linked immunosorbent assay: Individual CFAs

To determine IgY specific to individual adhesins represented on the adhesin-tip MEFA, anti-CFA adhesins and anti-EtpA IgY antibody titers were measured by ELISAs as previously described(4). Briefly, wells of 2HB 96-well microtiter plates (Thermo Scientific, Rochester, NY, USA) were coated, respectively, with 100 ng of each recombinant adhesin-tip subunit protein, i.e., CfaE (CFA/I), CooD (CS1), CotD (CS2), CstH (CS3), CsaE (CS4), CsfD (CS5), CssB (CS6), LngA (CS21) and EtpA. After blocking with 10% nonfat milk (Quality Biological, Inc., Gaithersburg MD), plates were incubated with 1:2 serially diluted chicken IgY samples, ranging from 1:200 to 1:51,200, at 37 °C for 1h and washed three times with PBS containing 0.05% Tween 20 (VWR International, Radnor, PA). Plates were incubated with HRP-conjugated goat anti-chicken IgY antibodies (1:10,000 dilution; Bethyl Laboratories, Montgomery, TX) at 37 °C for 1 h. TMB Microwell Peroxidase Substrate System (Kirkegaard & Perry Lab Inc., Gaithersburg, MD) was used to measure OD at 650 nm (OD_650_). IgY titers are presented as log transformation of the highest IgY sample dilution that produced an OD_650_ reading above 0.3 after subtraction of background, as described previously(4). Initial titers were determined on one egg from each of two hens; upon demonstration that titers were similar between eggs, all subsequent studies were carried out with pooled IgY from multiple eggs.

### Western immunoblotting

Prior to conducting adherence-inhibition studies to evaluate the anti-adherence impact of our anti-ETEC adhesin-tip MEFA IgY (hereafter, “active IgY”), we first performed western immunoblotting to verify that each target adhesin was in fact expressed in the growing cultures for each clinical outbreak-derived ETEC strain (**Table 2**). Briefly, each isolate was cultured under standard growth conditions in Luria-Bertani (LB) broth at 37°C overnight (16h) with aeration and normalized to an optical density (measured at a wavelength of 600 nm; OD_600_) of 0.6. Normalized liquid cultures were washed in 200 μl of PBS, sonicated for 30 s (Heat Systems Ultrasonics, Newtown, CT USA), and placed on ice for five minutes. Lysed cells were centrifuged at 10,000 × g for 20 minutes, and the supernatant was stored at -20°C.

**Table 2:** Enterotoxigenic *Escherichia coli* strains isolated from human patients presenting with diarrhea.

Strains	Relevant properties
E9034A	CS3 (18)
11829a	CFA/IV (19)
M447C4	CFA/II (20)
H10407	CFA/I (21)
31-10*	CFA/III (22)

ETEC strains used to determine impact of MEFA on clinical outbreak ETEC strains. “*” indicates strain encoding CFAs that are not represented on the ETEC adhesin-tip MEFA. CS: Coli surface antigens; CFA: Colonization factor antigens.

Each sample (10 μl) was run on a 12% polyacrylamide gel (NuSep, Germantown, MD, USA) using a Novex Mini-Cell (Invitrogen, Carlsbad, CA, USA). Samples were then transferred to nitrocellulose membranes (Azure Biosystems, Dublin, CA USA) and probed with either anti-MEFA or Unimmunized IgY. Blots were then incubated with HRP-conjugated goat anti-chicken IgY (1:2,500; ImmunoReagents, Inc., Raleigh, NC, USA) for one hour at room temperature, and visualized using Tetramethylbenzidine substrate (VWR International, Radnor, PA, USA), per manufacturer’s instructions.

### IgY performance – on-target effects

IgY extracted from pooled yolks collected between Weeks seven and 11 after the first immunization was evaluated for its performance against various ETEC strains’ adherence to mammalian cells in two laboratories. Caco2 colorectal adenocarcinoma cells were used in studies at Location 1; because of licensing considerations, screening studies at Location 2 were conducted in Vero (African green monkey kidney) cells, with subsequent verification in HT-29 human colorectal adenocarcinoma cells.

### ETEC adhesin-tip MEFA targeted IgY: Antibody adherence inhibition assays

#### Impact of anti-ETEC adhesin-tip MEFA IgY on MEFA design ETEC strains

To evaluate anti-MEFA IgY neutralizing activities against bacterial adherence, we first examined the ability of anti-MEFA IgY WSF to inhibit adherence of ETEC strains used in design of the adhesin-tip MEFA (**Table 1**) to Caco-2 cells (HTB-37™; American Type Culture Collection, Manassas, VA, USA), as described previously(4). Briefly, ETEC isolates encoding various adhesin-tip MEFA epitopes, i.e., CFA/I, CS1, CS2, CS3, CS4/CS6, CS5/CS6, CS6, CS21 or EtpA, were grown in liquid culture to 10^6^ CFU/ml and pre-treated with mannose (4% v/v). For each strain, three biological replicates were incubated with 15 µl of anti-MEFA or unimmunized IgY samples (10 mg IgY/ml PBS) at 24 °C for 30 min with aeration. Each IgY sample/bacteria mixture was normalized to 300 µl with Eagle’s Minimum Essential Medium (American Type Culture Collection, Manassas, VA, USA), for a final IgY concentration of 0.5 µg/µl. The IgY/bacteria mixture was then added to 10^5^ Caco-2 cells in a 48-well plate at a multiplicity of infection ratio of 10 bacterial cells per Caco-2 cell. After incubation in a 5% CO_2_ incubator at 37 °C for 1 h, Caco-2 cells were rinsed with PBS (to remove non-adherent bacteria) and dislodged with 0.5% Triton X-100 (Sigma-Aldrich, St. Louis, MO, USA). *E. coli* adherent to Caco-2 cells were collected, serially diluted (1:10) three times. Each dilution was spread on LB solid media plates (MP Biomedicals, Solon, OH, USA) and incubated overnight (16 h) at 37°C. The following day, visible colonies were counted as colony forming units (CFU).

#### Impact of anti-ETEC adhesin-tip MEFA IgY on clinical outbreak ETEC strains

To initially screen for adherence-inhibition properties of IgY against clinically obtained ETEC strains expressing CFA/II, CFA/III, CFA/IV, or CS3 (**Table 2**), we employed methods similar to those used for MEFA-design strains, with slight modifications(4). All strains were prepared under standard growth conditions in LB broth (VWR Life Science) at 37°C with aeration overnight and normalized to an OD_650_ range of 0.6 using fresh LB broth. Bacteria were pre-treated with 4% (w/v) mannose and incubated with 45 µl of anti-adhesin-tip MEFA IgY, unimmunized IgY, (10 mg/ml), or no IgY. After incubation at room temperature for 30 min with aeration, each IgY/bacteria mixture was normalized to 900 µl with PBS (40% v/v), and 180 µl of the bacteria/IgY mixture was added to 10^5^ Vero cells (CCL-81™; American Type Culture Collection, Manassas, VA, USA) in each well of a 48-well microtiter plate. Vero cells were incubated and processed following the techniques described above to determine CFU count.

Because Vero cells are not intestinal epithelial cells, we elected to validate the impact of anti-ETEC adhesin-tip MEFA directed IgY on ETEC adherence to human gut cells in culture. To do so we repeated the adherence-inhibition assay in the five clinical outbreak strains using human HT-29 colorectal adenocarcinoma cells (ATCC HTB-38™). All other aspects of this assay were identical to those used above.

### IgY Performance: Off-target concerns

To address potential concerns about the impact of anti-ETEC adhesin-tip MEFA directed IgY on non-target bacteria, we examined growth and adherence-inhibition characteristics of the IgY through growth and adherence inhibition assays.

### Growth inhibition assays

To determine the impact of anti-adhesin-tip MEFA IgY on bacterial viability, growth inhibition assays were conducted by OD method as previously described (23), with minor variations. Three biological replicates of ETEC isolates encoding CFA/II, CFA/III, CFA/IV, or CS3 (**Table 1**) and a commensal *E. coli* strain that does not encode any of the adhesins included on MEFA (BL21; Genotype: F^-^, *omp*T, *hsd*S_B_ (r_B_–, m_B_–), *dcm*, *gal*, λ(DE3), pLysS, Cm^r^; Promega, Madison, WI, USA) were evaluated. Isolates were grown in LB broth overnight at 37°C with aeration and normalized to an OD_650_ of 0.06 using fresh LB broth. Anti-MEFA IgY or unimmunized IgY (0.4 mg) in 40 μl of PBS was added to each well of a 96-well microtiter plate. PBS was used as a blank control. Ciprofloxacin hydrochloride in PBS (1 ng/μl) was used as negative assay control, and bacterial isolates with no IgY or antibiotic supplementation was used as positive control. A total of 260 μl of LB broth was added to each well and inoculated with 20 μl of each normalized culture, respectively. Cultures were incubated at 37°C with aeration for up to 18 h, and OD_650_ was recorded hourly using a THERMOmax microplate reader (Molecular Devices, Sunnyvale, California, USA). Growth rates were presented as the maximum hourly change in OD_650_ for each isolate across three technical replicates.

### Non-ETEC adherence inhibition assays

To determine the impact of anti-adhesin-tip MEFA IgY on adherence of non-ETEC bacterial strains to human cells in culture, carried out adherence inhibition studies against three nonpathogenic bacterial strains: *Lactobacillus casei (ATCC^®^ 393™)* [a probiotic bacterium recently shown to have some anti-diarrheal effects in children) (24), *E. coli* HS (a human commensal strain (25)], and *E. coli* DH5α, a K12-derived laboratory strain (26). Methods were identical to those previously described for ETEC strains, with the exception that human cervical adenocarcinoma cells (HeLa, ATCC CRM-CCL-2™), commonly used in infectious disease research, were used in all three assays.

### Statistical analysis

Adhesion inhibition assays are presented as a hybrid box plot and kernel density plot, i.e., a violin plot, showing the result of three biological replicates performed in technical triplicate (n=9). Boxes represent the interquartile range (IQR) with the median shown as the center bar of each sample group. Whiskers represent 1.5 times the IQR. P-value, by two-sample t-test method, and 95% confidence intervals were calculated using the R software package EnvStats v.2.3.1 (27).

## Results

### Adhesin-tip MEFA is a strong immunogen in laying hens

ELISA of IgY production in hens immunized with adhesin-tip MEFA showed undetectable MEFA specific antibody production on the day prior to the first immunization, with detectable anti-MEFA IgY by two weeks post-immunization, achieving titers of 1:524,288 (1:2^19^) after nine weeks. Furthermore, production of anti-adhesin-tip MEFA IgY was sustained at or above these titers until at least 24 weeks, when the recording period ended. That is, ELISA titers were 1:2^19^ from weeks 9 to 15, rising to 1:2^20^ at weeks 16-19, and returning to 2^19^ at week 24 (**Figure S1**). By contrast, ELISA of both unimmunized and sham-immunized hens IgY revealed no detectable antigen-specific antibodies. Therefore, the control condition is hereafter referred to as “Unimmunized IgY.”

The purity of the extracted IgY was demonstrated by SDS-PAGE, revealing clear bands at molecular weights expected for purified IgY, with a heavy chain at 68 kD and light chain 24 kD, respectively, and little other protein material (**Figure S2**). The yield of IgY (total protein) as determined by BCA assay, was approximately 10 mg/ml; based on the lack of other proteinaceous material on SDS-PAGE, all measured protein was considered to be IgY. IgY collected from pooled yolks during post-immunization weeks seven to 11, with anti-adhesin-tip MEFA titers of 1:2^19^ (1:524,288) was used in all subsequent analyses.

**Figure 1:**
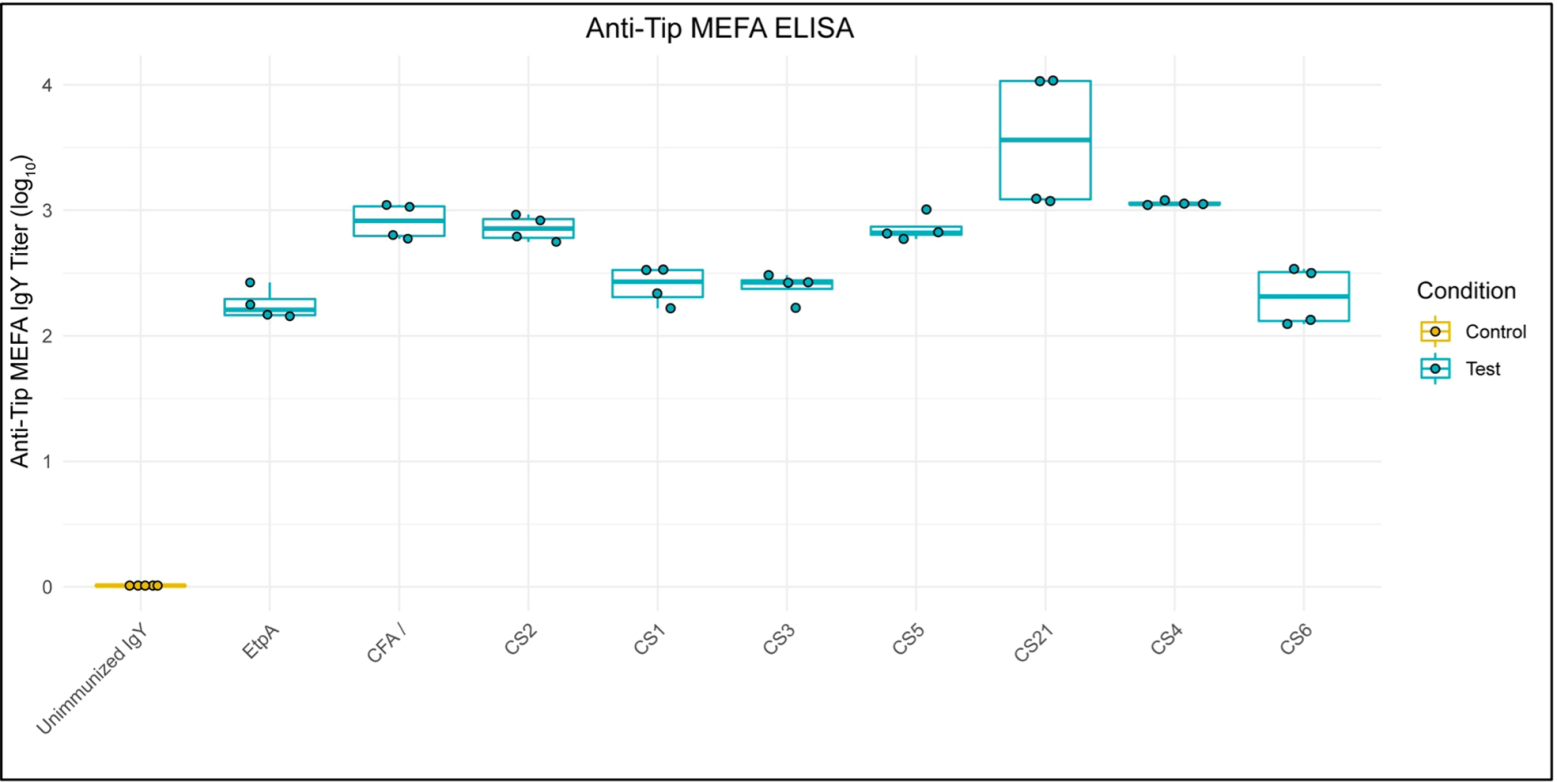
Anti-ETEC Adhesin-Tip MEFA IgY Binds Individual MEFA Adhesins. Box and whisker plots show ELISA results of anti-adhesin-tip MEFA IgY binding to each of nine individual adhesin tip epitopes (log_10_). Unimmunized IgY demonstrated undetectable ELISA signal against each epitope and is presented as a normalized control group. Boxes represent interquartile range (IQR) with median shown as center bar of each sample group. Whiskers represent 1.5 times the IQR. Gold, Unimmunized IgY; teal, anti-MEFA IgY.

ELISA showed anti-adhesin-tip MEFA IgY (log_10_) titers of at least two, and as high as 3.5 against all individual adhesins represented on the MEFA, reflecting the varying immunogenicity of each adhesin-tip moiety on the MEFA, while titers were non-detectable against any of the MEFA adhesins when tested against unimmunized IgY (**Figure 1**).

### Anti-ETEC adhesin-tip MEFA IgY inhibits ETEC adherence to mammalian cells *in vitro*

**Figure 2:**
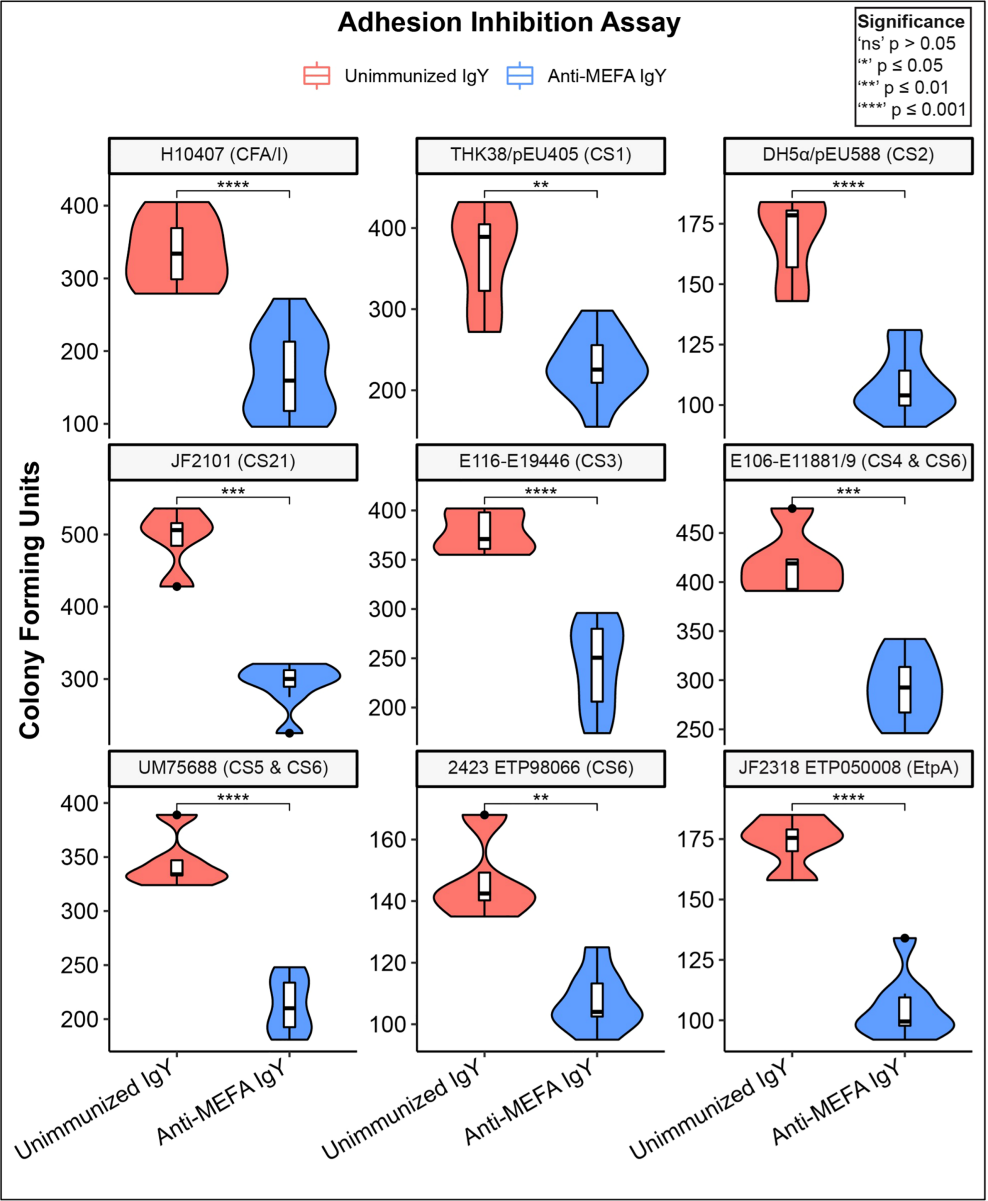
Adherence inhibition of MEFA derivation ETEC strains by anti-MEFA IgY. Violin plots of adherence-inhibition assay for ETEC strains used in derivation of the adhesin-tip MEFA. Degree of adhesion inhibition to Caco2 cells by comparison with control (unimmunized) IgY is shown; significant reduction (P < 0.01) in adhesion was demonstrated across all strains. Boxes represent interquartile range (IQR) with median shown as center bar of each sample group. Whiskers represent 1.5 times the IQR. P-value, by two-sample t-test method, and 95% confidence interval (CI) was calculated using R software package EnvStats (v.2.3.1) (27). Red, unimmunized IgY; Blue, anti-MEFA IgY.

Western blotting demonstrated expression by outbreak-associated strains of the adhesins listed in **Table 2**, when the organisms were grown in LB broth **(** **Figure S3** **)**. IgY extracted from pooled yolks of adhesin-tip MEFA-immunized hens inhibited adherence of ETEC strains grown in LB to mammalian cells. **Figure 2** shows adherence-inhibition results for the nine strains used in the development of the MEFA listed in **Table 1**. Individual CFU counts vary between strains; the comparison being made is within-strain by treatment condition. Adherence of all MEFA derivation ETEC strains examined in this study was inhibited by anti-MEFA IgY (P < 0.01).

To determine if adhesin-tip MEFA IgY is capable of inhibiting adherence of clinically relevant ETEC strains in addition to those used in design of the MEFA, we initially screened for adherence inhibition in Vero cells of eight isolates from clinical ETEC outbreaks, including strains bearing CFA/CSs represented on the adhesin-tip MEFA, as well as two strains bearing CFA/III, not represented on the MEFA (strains 31-10 and MP215-1). Results demonstrated significant inhibition (P < 0.05) of adhesion, compared with the No IgY condition for all strains except MP215-1 (**Figure S4**). Inhibition by unimmunized IgY compared with the “No IgY” condition was demonstrated to be significant in four of the eight strains. However, in all strains, with exception of MP215-1, the active IgY produced significantly greater (P < 0.05) inhibition than did unimmunized IgY.

**Figure 3:**
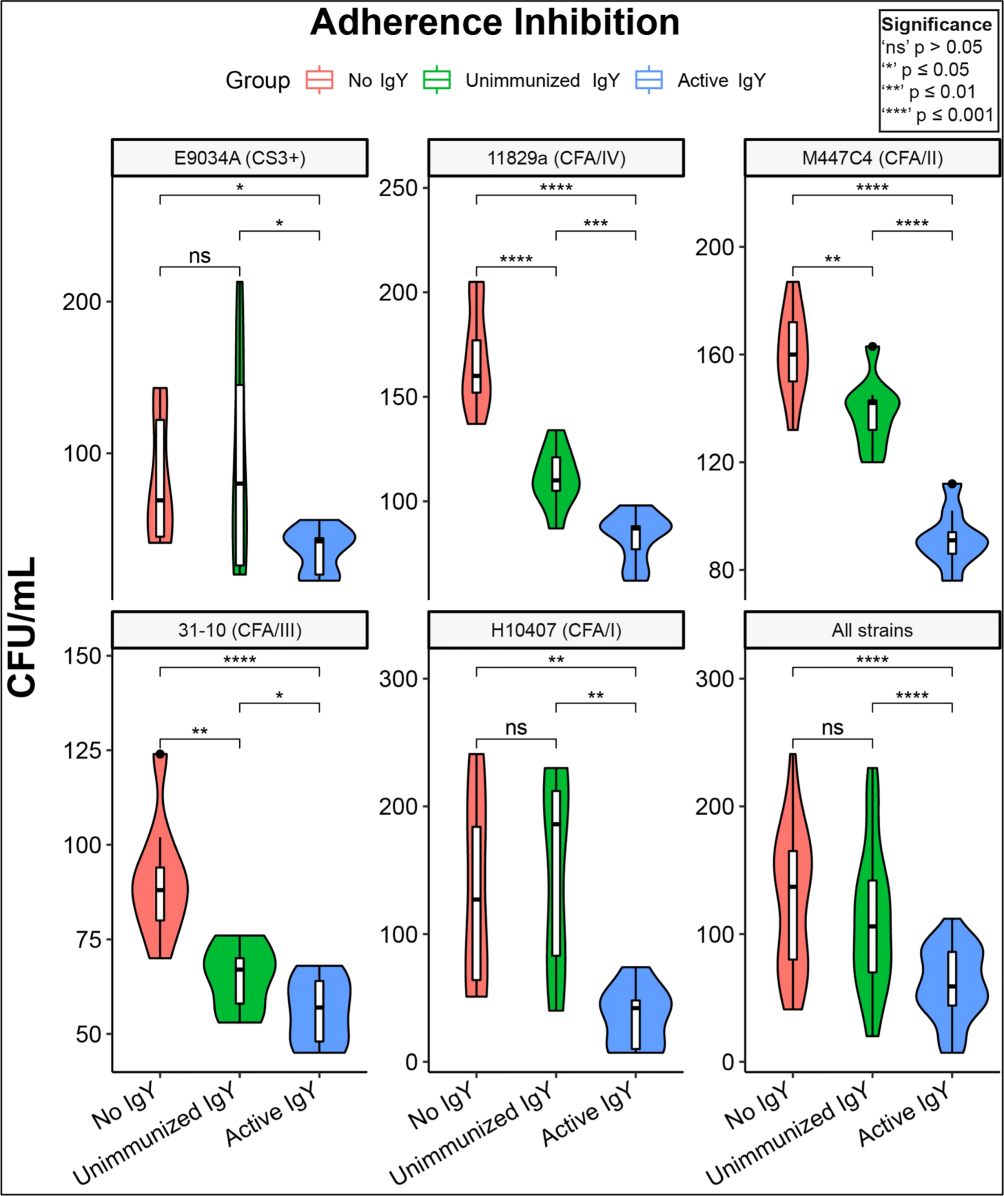
Adhesion inhibition of clinical ETEC isolates by anti-MEFA IgY. Violin plots of adherence-inhibition assay for the five clinical ETEC strains tested in HT-29 cell cultures; the lower right panel shows the aggregate effect on all strains. All five outbreak-associated strains demonstrated significant (P<0.5) reduction in adhesion by active anti-MEFA IgY in comparison with both “No IgY” and “Unimmunized IgY” conditions. Three of the five strains also demonstrate modest but significant adherence inhibition by Unimmunized IgY compared with No IgY. Boxes represent interquartile range (IQR) with median shown as center bar of each sample group. Whiskers represent 1.5 times the IQR. P-value, by two-sample t-test method, and 95% confidence interval (CI) was calculated using R software package EnvStats (v.2.3.1) (36). Red, no IgY; green, unimmunized IgY; blue, anti-MEFA IgY.

To verify adherence inhibition in a human gut cell line, we repeated the adherence inhibition study using HT-29 human adenocarcinoma cells. Again, in all cases, anti-adhesin-tip MEFA IgY significantly decreased adhesion in comparison with both unimmunized and No IgY conditions (**Figure 3**). Slight but significant inhibition of adherence relative to the no IgY condition was seen in the unimmunized IgY condition against three of the five strains tested. However, in all strains individual inhibition was significantly greater in the active IgY groups compared to that of the unimmunized IgY groups.

### IgY Effects on adherence of representative commensals

Anti-ETEC adhesin-tip MEFA-directed IgY had no detectable impact on adherence of representative non-ETEC commensal strains *L. casei, E. coli* HS, or *E. coli* DH5α, in comparison to the “no IgY” and “Unimmunized IgY” conditions (**Figure S5**).

### IgY effects on bacterial growth

**Figure 4:**
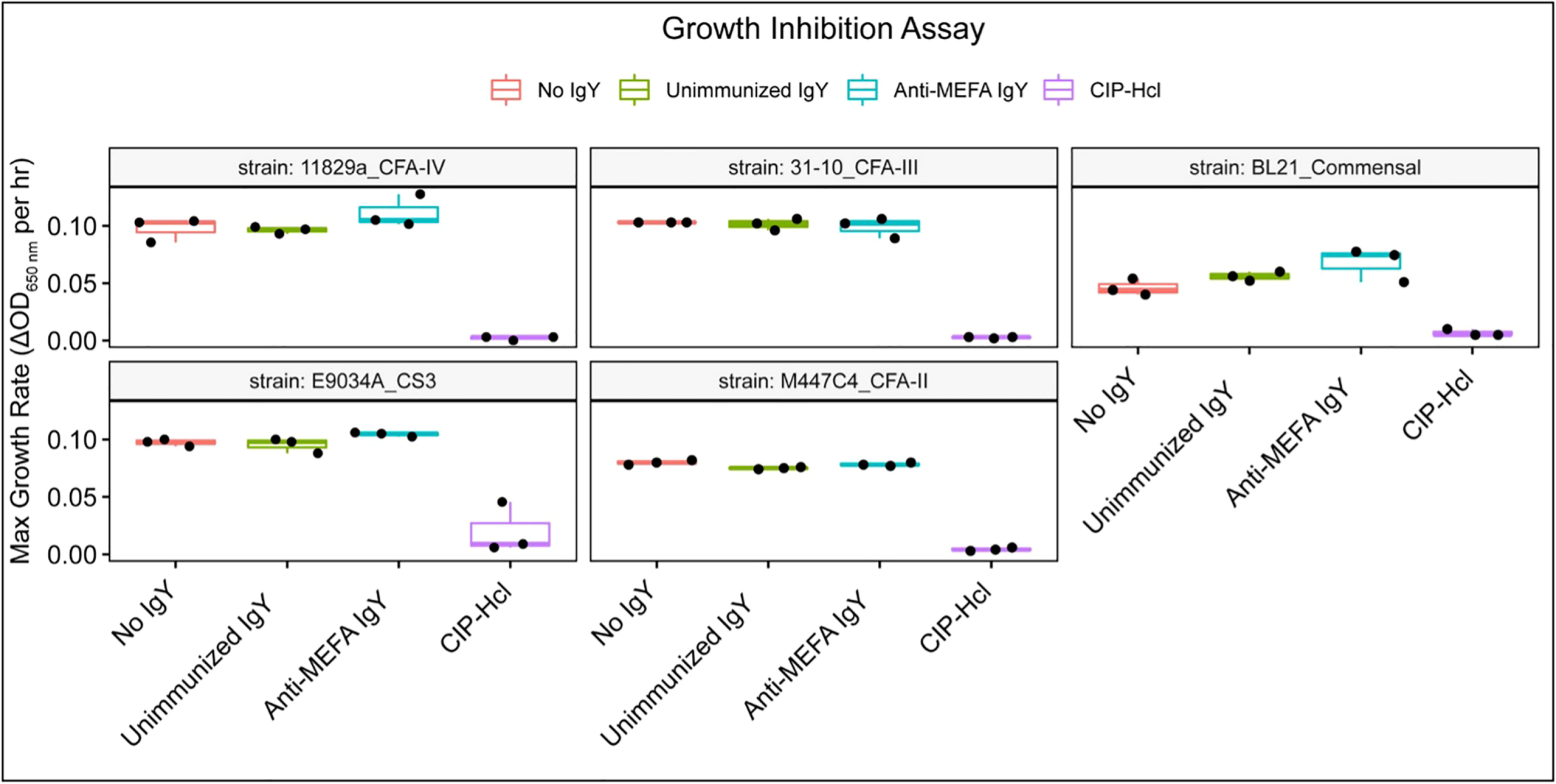
Growth inhibition assay. Box and whisker plots of the maximum growth rate, defined as change in OD650 per hour, for each isolate across three technical replicates. Growth was inhibited in the presence of ciprofloxacin hydrochloride. However, the detectable impact of IgY on growth was minimal. Boxes represent interquartile range (IQR) with median shown as center bar of each sample group. Whiskers represent 1.5 times the IQR. Red, no IgY; green, unimmunized IgY; blue, anti-MEFA IgY; purple, ciprofloxacin hydrochloride (CIP-HCl, 1 ng/ml).

Growth of all isolates was inhibited by ciprofloxacin hydrochloride, as expected; however, there was no detectable effect of anti-ETEC adhesin-tip MEFA IgY on bacterial growth for any of the eight clinical isolates or of the commensal strain examined under the growth conditions included here (**Figure 4**).

## Discussion

The use of passive antibodies as prophylaxis against diarrheal illness may be a promising complement to vaccines, which have yet to receive regulatory approval or reach markets. Some populations, such as short-term travelers, may prefer an alternative to immunizations, particularly those requiring booster administration. Similarly, for infant and immunocompromised populations who may not respond to vaccines, an oral IgY formulation could be a useful short-term strategy.

Herein, we explored aspects of avian IgY raised against multiple adhesin tip virulence factors of ETEC, a leading cause of diarrhea globally(1). The adhesin-tip MEFA employed uses a backbone protein and substitutes surface-exposed peptides of the backbone with epitopes of foreign antigens, suggested previously to improve construct immunogenicity in murine models(4). We sought to determine whether this MEFA had utility in production of avian IgY as a potential source for temporary passive immunization against multiple strains of ETEC in humans *in vitro*.

Results showed that anti-ETEC adhesin-tip MEFA polyclonal IgY significantly reduced adhesion of multiple laboratory and outbreak-associated strains of ETEC to mammalian cells in culture (**Figures 2**, **3**). Adhesion is a prerequisite for elaboration of the diarrhea-inducing heat-stable toxins (ST) and heat-labile toxins (LT) that ultimately induce fluid secretion from the ileal mucosa by modulation of cAMP and cyclic guanosine monophosphate (cGMP) (28). Further studies are planned to evaluate the impact on cyclic nucleotides of the anti-adhesin-tip MEFA on human intestinal cells in culture. A reduction in cyclic nucleotide concentrations in anti-adhesin-tip MEFA IgY-treated cells would suggest that inhibiting ETEC adhesion has a direct physiologic effect on the host cells and may be predictive of reduced fluid losses *in vivo*.

Because we examined IgY-induced adhesion inhibition against both ETEC strains used in derivation of the MEFA and against clinical isolates obtained from disease outbreaks, we believe that this IgY preparation could have general utility in prophylaxis of TD and other enteric diseases. We demonstrated adhesion inhibition against one of two ETEC isolates that do not encode adhesins included on the MEFA (Strain 31-10, bearing CFA/III), suggesting potentially beneficial cross-reactivity for broad range clinical applicability. Further studies are underway to clarify this effect.

In some cases, we observed inhibition by non-specific (unimmunized) IgY against ETEC strains (**Figures 3** **,** **S4**). We suspect that this indicates non-specific activity of avian antibodies in general; alternatively, this finding could arise from anti-adhesin IgY present in our laying hens, derived from environmental exposure to non-ETEC *E. coli.* However, under all experimental conditions, inhibition by target specific IgY was significantly greater (P < 0.05) than that of non-specific antibodies. In the future, use of specific pathogen-free (SPF) hens may clarify this issue. Further evidence for a mild, nonspecific effect of unimmunized IgY comes from findings in a 2012 study of anti-rotavirus IgY in gnotobiotic pigs (29), in which a slight clinical reduction in diarrheal severity was observed in animals treated with UI IgY; however, UI-treated animals’ diarrhea was still significantly more severe than that of pigs receiving rotavirus-specific IgY treatment. These findings suggest that properties inherent to IgY in general, rather than prior exposure of hens to the antigen, account for this intermediate level of effect.

In practice, there remains the possibility that other pathogenic *E. coli* isolates may express additional virulence factors or are not affected by the adhesin-tip MEFA IgY, or that antigenic drift may produce novel epitopes not recognized by IgY targeting this specific adhesin-tip MEFA. One advantage of using rapidly-developed and -produced IgY is that as new antigenic targets are identified, new IgY formulations can be generated and tested within a few weeks. However, studies of IgY targeting LT and ST should also be considered as a means of providing immunoprophylaxis over and above that provided by targeting adhesins; there is historical evidence of synergy between anti-bacterial and anti-toxin antibodies in both ETEC and toxigenic *Vibrio cholerae*, which produces cholera toxin, responsible for causing acute diarrhea associated with cholera (30, 31). If successful, future formulations for human prophylaxis could include such antibodies as well to enhance the prophylactic effect against multiple antigens involved in ETEC diarrheal pathogenesis. Of interest, a human vaccine preparation using both an adhesin-based MEFA and a toxoid fusion moiety has recently been shown to be effective at preventing ETEC-induced diarrhea in a porcine disease model (32) and ETEC colonization of rabbit small intestine(6).

We found no detectable impact of the IgY used here on growth of the *E. coli* strains examined in this study, and we speculate that adhesin-targeted IgY may have limited impact on viability of the ileal or colonic flora, but can alter targeted function, i.e., adhesion to ileal mucosal cells. However, further investigation of bacteriostatic properties, if any, of IgY *in vitro* are needed to confirm these observations.

Existing passive immunity-conferring preparations, such as those containing bovine colostrum, target only single ETEC strains such as H10407(10). A broader-spectrum array of antibodies such as those described herein may help to maximize prophylactic coverage.

The number of hens used for IgY production in this study is similar to that used by prior researchers for *in vitro* studies of analytical quantities of IgY (33–35). However, IgY can readily be produced in large volumes, and new immunization techniques including viral vectors and *in ovo* vaccination can enhance IgY production and smooth the logistics of preparing kilogram quantities of material (14, 36). Once extracted and lyophilized, IgY can be stored at temperatures up to 60° C for lengthy periods (14). Together, these factors suggest scalable production is feasible, and that packaging, transport, and distribution issues will be simpler than those associated with most vaccine candidates.

While we demonstrated significant adhesion-inhibition of all ETEC strains tested, including strains with adhesins not represented on the MEFA, the possibility remains that still other disease-inducing strains may evade such inhibition and produce disease. This study did not include *in vivo* evaluations, limiting the strength and applicability of its conclusions. Future studies should examine impact on additional strains and genotypes of these pathogens; the rapid production and evaluation of IgY may provide a basis for future modifications of any prophylactic formulation. Finally, further work should be performed to better characterize the binding interactions between the anti-adhesin-tip MEFA IgY and its MEFA target, as well as between the IgY and individual adhesins.

## Conclusions

Avian immunoglobulins (IgY) are produced readily against an adhesin-tip multiepitope fusion antigen (MEFA) at high and sustained titers. This IgY has strong inhibitory effects on ETEC adherence, but no detectable adverse effects on bacterial growth of target or commensal *E. coli* strains. Collectively, these findings suggest that anti-ETEC adhesin-tip MEFA IgY might be effective at preventing ETEC adherence, a critical first step in host colonization, and subsequent toxin delivery by one of the leading causes of childhood and travelers’ diarrhea. Effects of a prophylactic formulation of this IgY will be tested in suitable animal models. Passive oral immunization with target specific IgY may provide an appealing prophylactic approach until vaccines are developed with the desired efficacy and may provide a useful alternative for travelers reluctant to receive systemic vaccines.

## Data availability statement

The raw data supporting the conclusions of this article will be made available by the authors, without undue reservation.

## Ethics statement

Protocols for hen maintenance and immunization were approved by the Scaled Microbiomics, LLC Animal Use and Care Committee (approval number 19-111 01-TD).

## Author contributions

KB, HS, NI, CA, WZ, DS, and JGo contributed to study design and execution. KB, LG, JN, JGe, and JGo contributed to manuscript development, interpretation of results, and data presentation. All authors contributed to the article and approved the submitted version.

## Acknowledgments

The authors thank Dr. Jorge Giron (Translational Genomics Research Institute, an affiliate of City of Hope, Phoenix, AZ), who kindly provided the *Escherichia coli* outbreak-associated isolates used in this study. David Myers (Rohrersville, MD) provided care of the laying hens used in this study.

## Conflict of interest

Authors CA, NI, and JG were employees of Scaled Microbiomics at the time this work was carried out and holdequity in the company. JN holds equity in Scaled Microbiomics, LLC.

The remaining authors declare that the research was conducted in the absence of any commercial or financial relationships that could be construed as a potential conflict of interest.

The authors declare that this study received funding from Scaled Microbiomics, LLC, which holds a pending patent on the use of IgY in the prophylaxis and treatment of Human Norovirus and Enterotoxigenic E. coli infection. The funder had the following involvement in the study: Determination of study priorities including IgY targets.

## Publisher’s note

All claims expressed in this article are solely those of the authors and do not necessarily represent those of their affiliated organizations, or those of the publisher, the editors and the reviewers. Any product that may be evaluated in this article, or claim that may be made by its manufacturer, is not guaranteed or endorsed by the publisher.
