# Supplementary material for: Feasibility of avian antibodies as prophylaxis against enterotoxigenic escherichia coli colonization
Source: Front Immunol. 2022 Oct 19;13:1011200. doi: 10.3389/fimmu.2022.1011200 (PMC9627289; doi:10.3389/fimmu.2022.1011200)
Supplement: Supplementary file 1 [file DataSheet_1.pdf]

**Supplementary Figures for ETEC IgY paper**

**Figure S1**

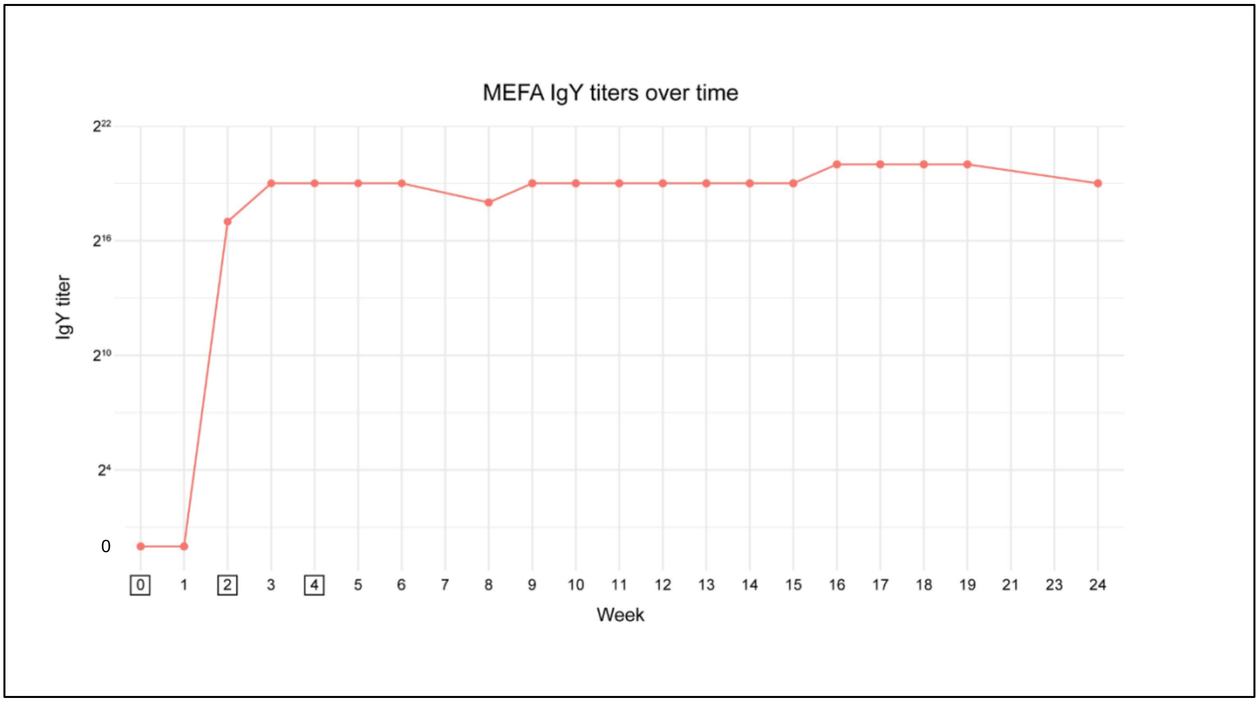


***Figure S1.*** *ELISA titers (log2) of IgY targeting ETEC adhesin-tip MEFA over 24 weeks are shown as line plots. Immunizations were given at weeks 0, 2, and 4.*

**Figure S2**

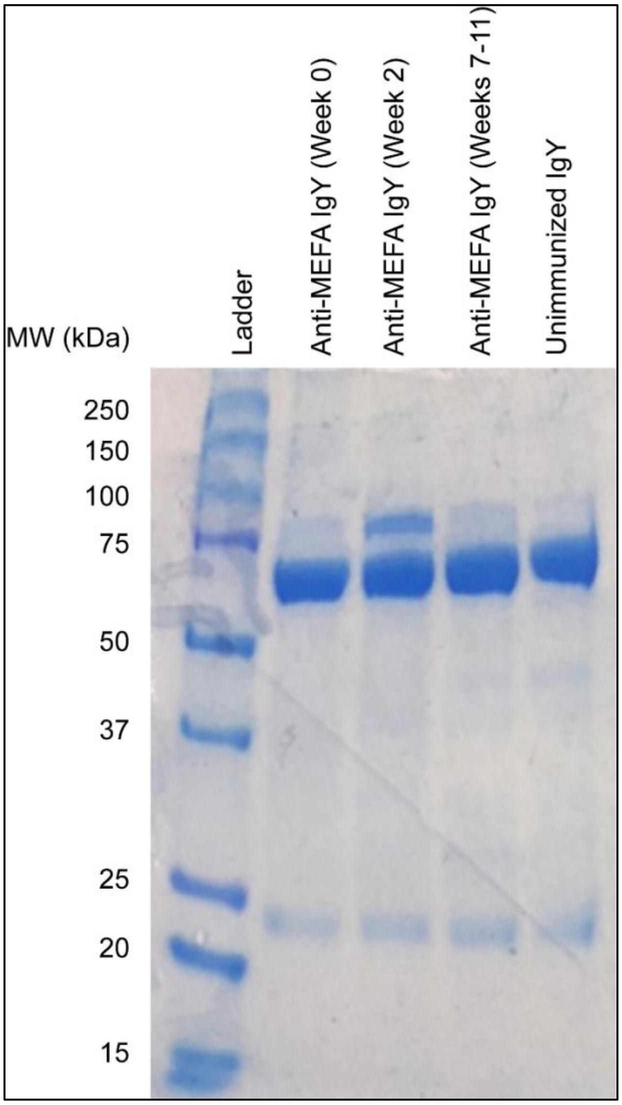


***Figure S2:*** *Image of SDS-PAGE conducted under reducing conditions. Lane loading scheme is as labeled. Precision Plus Dual Color Pre-stained protein standard (10-250kDa; Bio-Rad, Hercules, CA, USA) was used as a molecular weight marker. IgY heavy and light chains show characteristic bands at 68 and 24 kDa, respectively. IgY from unimmunized hens was used as control.*

**Figure S3**

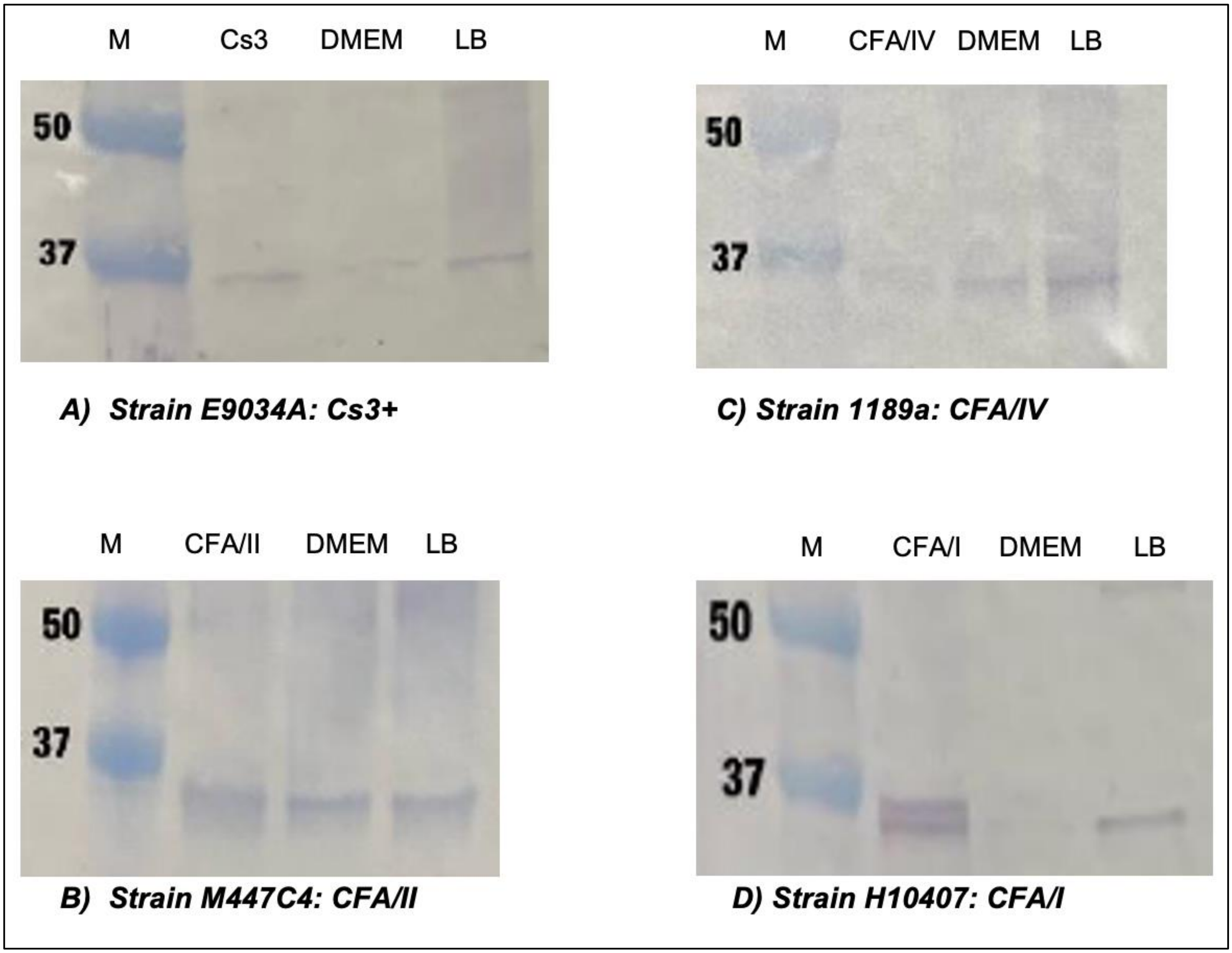


***Figure S3:*** *Western blot images of lysed ETEC strains treated with anti-ETEC adhesin-tip MEFA, indicating expression of each listed adhesin. M: Molecular weight marker; Lane 1: Purified adhesin alone; Lane 2: lysed ETEC strains grown in DMEM culture medium; Lane 3: Lysed ETEC strains grown in LB culture medium. NB: No blot is shown for ETEC strain 31-10, expressing CFA/III; this adhesin is absent from the immunizing adhesin-tip MEFA.*

**Figure S4**

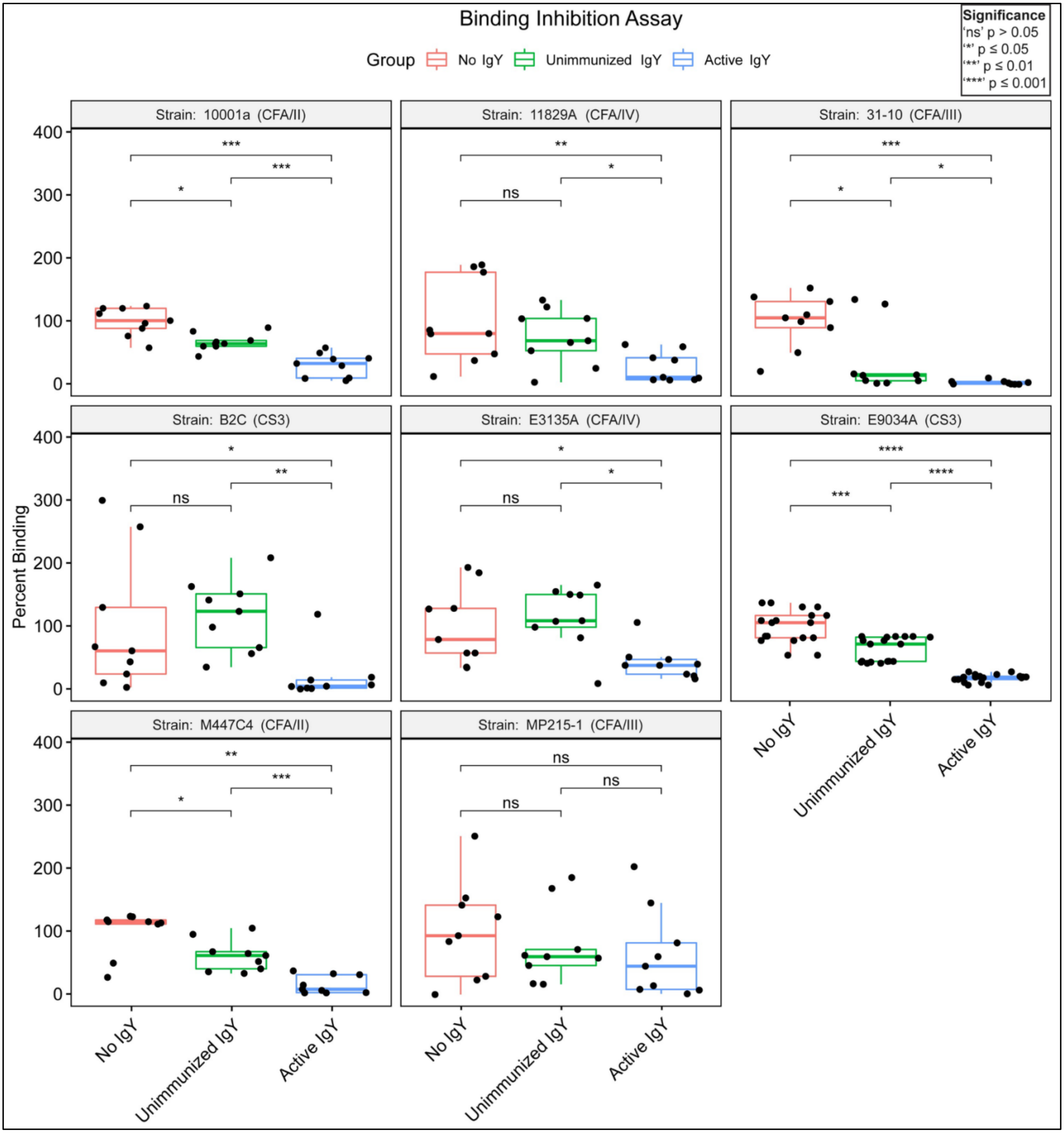


*__Figure S4.__ Box and whisker plots of adherence-inhibition assay for clinical ETEC strains on Vero cells in culture. With the exception of MP215-1, all strains show significant ($P \leq 0.05$) inhibition of adhesion to Vero cells by anti-MEFA IgY in comparison with both "No IgY" and Unimmunized IgY conditions; most strains also*

*demonstrate some degree of inhibition by Unimmunized IgY compared with No IgY, suggesting a non-specific inhibitory effect of IgY proteins in general. "Percent binding" is displayed as the normalized average of control IgY. Boxes represent interquartile range (IQR) with median shown as center bar of each sample group. Whiskers represent 1.5 times the IQR. P-value, by two-sample t-test method, and 95% confidence interval (CI) was calculated using R software package EnvStats (v.2.3.1) (Millard, 2013). Coral, no IgY; green, unimmunized IgY; blue, anti-MEFA IgY.*

**Figure S5**

**A:** *Lactobacillus casei*

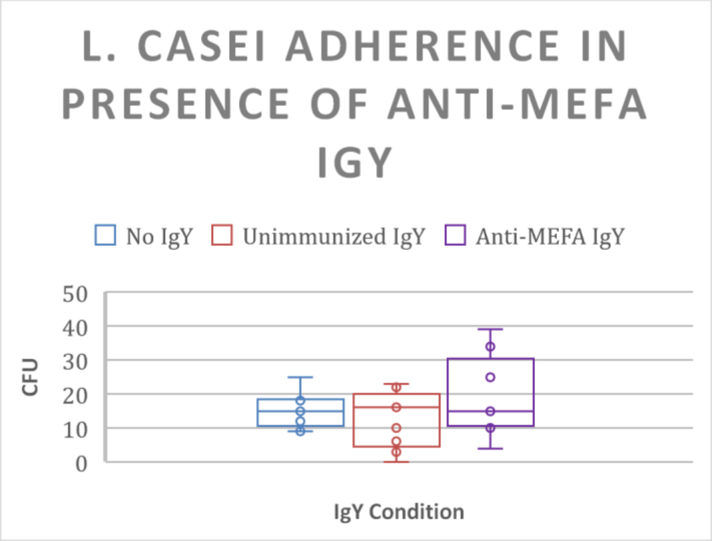


**B:** *E. coli* DH5alpha

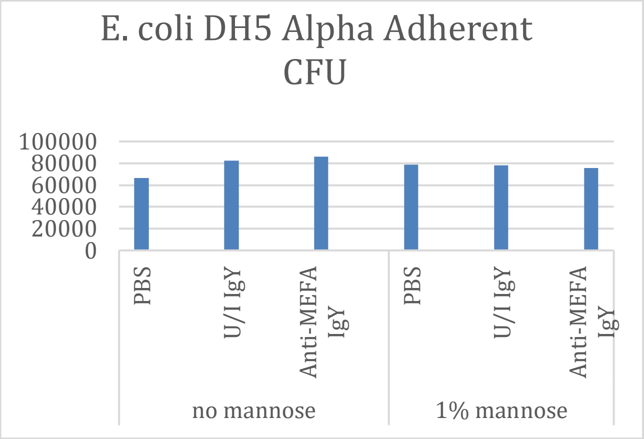


**C:** *E. coli* HS

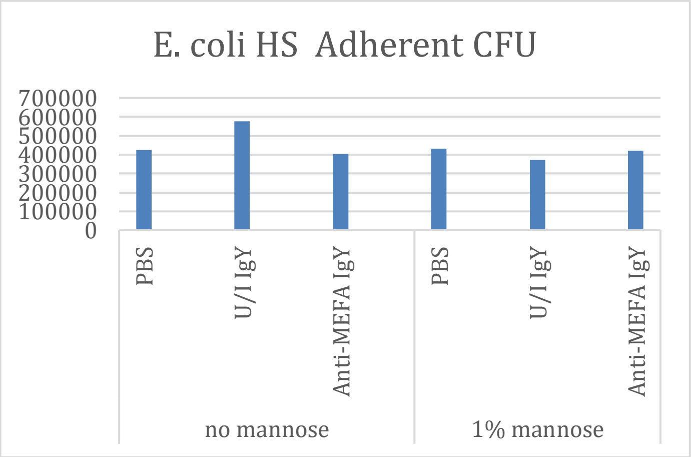


***Figure S5:*** *Adherence of selected commensal bacterial strains to HeLa cells under treatment with No IgY/PBS, Unimmunized IgY, and active anti-adhesin-tip MEFA IgY. All tests non-significant (P>0.05).*
